# Only collective action can fight off winter influenza outbreaks

**DOI:** 10.1016/j.eclinm.2018.12.004

**Published:** 2018-12-07

Nobody likes to get sick, so why are many people averse to receiving the influenza vaccine and whose responsibility is it to be vaccinated?

Discussions of influenza vaccination often focus on the most at-risk groups of the population. These include adults older than 65 years, children younger than 5 years, and pregnant women. Among these groups the greatest rate of vaccine uptake is in people older than 65 years: approximately 72·6% in 2017–18, up from the previous year. The proportional uptake is lower for the other groups: 47·2% of pregnant women opted to receive vaccination in 2017–18 (data available up to Jan 31), although this was greater than the 44·9% uptake in 2016–17. Pilot studies offering vaccination to children in school years 1–4 in England showed an uptake of 59·5% in 2017–18 [1]. This uptake greatly varied by region, ranging from 47·8% to 70·7%, with the lowest uptake recorded in London. In the context of the broad population (aged between 6 months and 65 years, excluding pregnant women), uptake does not look better. In this group, the rate of vaccination for 2017–18 was 48·9%, which is surprisingly low for an easily available and well publicised intervention.

Influenza incidence has fallen since the 2009 H1N1 influenza pandemic, and for the past 4 years rates have remained at moderate levels [1]. In the UK, much of the credit for the lower incidence is due to the activity of the Influenza Surveillance Team at Public Health England’s National Infection Service. However, data from last year raise concern. The UK Severe Influenza Surveillance Systems (USISS) sentinel scheme reported increases in hospitalised confirmed influenza in 2017–18 versus 2016–17. The 2017–18 incidence is the highest noted in the 5-year surveillance history of the scheme. The USISS mandatory surveillance scheme assesses intensive-care and high dependency units in the UK, which tend to receive the most serious influenza cases. The USISS analysis reported 320 deaths in England in 2017–18 compared with 112 in 2016–17 and 166 in 2015–16 [1]. These reports show that influenza incidence is on the rise and highlight that this is far from being an easily managed illness. This situation is, in part, attributable to the general public’s attitude towards the infection.

The European Commission’s state of vaccine confidence report reveals some interesting insights into public attitudes. Although confidence in vaccination has increased over the past 5 years, nearly 15% of people still feel that the seasonal influenza vaccine is unsafe and 20% of people did not feel that the influenza vaccine was at all important [2]. Almost 20% of respondents in the UK felt vaccinations were incompatible with their religious beliefs. This finding needs further analysis and emphasises the need to consider places of worship for these groups as targets for education.

A significant factor affecting the uptake of vaccination is a misunderstanding of the exact morbidity of influenza, and the burden it represents for society. Individuals often confuse what is colloquially referred to as the flu with the more serious, potentially life-threatening effects of influenza. This confusion leads many people to assume they will be able to simply deal with a bout of influenza without considering the potential burden on health-care resources and the potential for spread to more vulnerable groups. Indeed, during the 2017–18 peak yearly window for influenza, the period saw vast increases in NHS burden in England. General practitioner consultations in 2017–18 peaked at over 40 h per 100 000 population per week—in the 3 previous years this did not rise above 20 h. Similar effects were reported in the use of the NHS 111 emergency service for influenza-like illness and out-of-hours consultations. These issues were mirrored across the UK. Additionally, several studies showed the value of vaccinating low-risk populations (i.e., children, adolescents, and young adults) to protect the more vulnerable individuals [3], [4], [5]. Providing education on the benefits of herd immunity, as well as vaccination in general, would possibly improve vaccination uptake [6].

**Figure:**
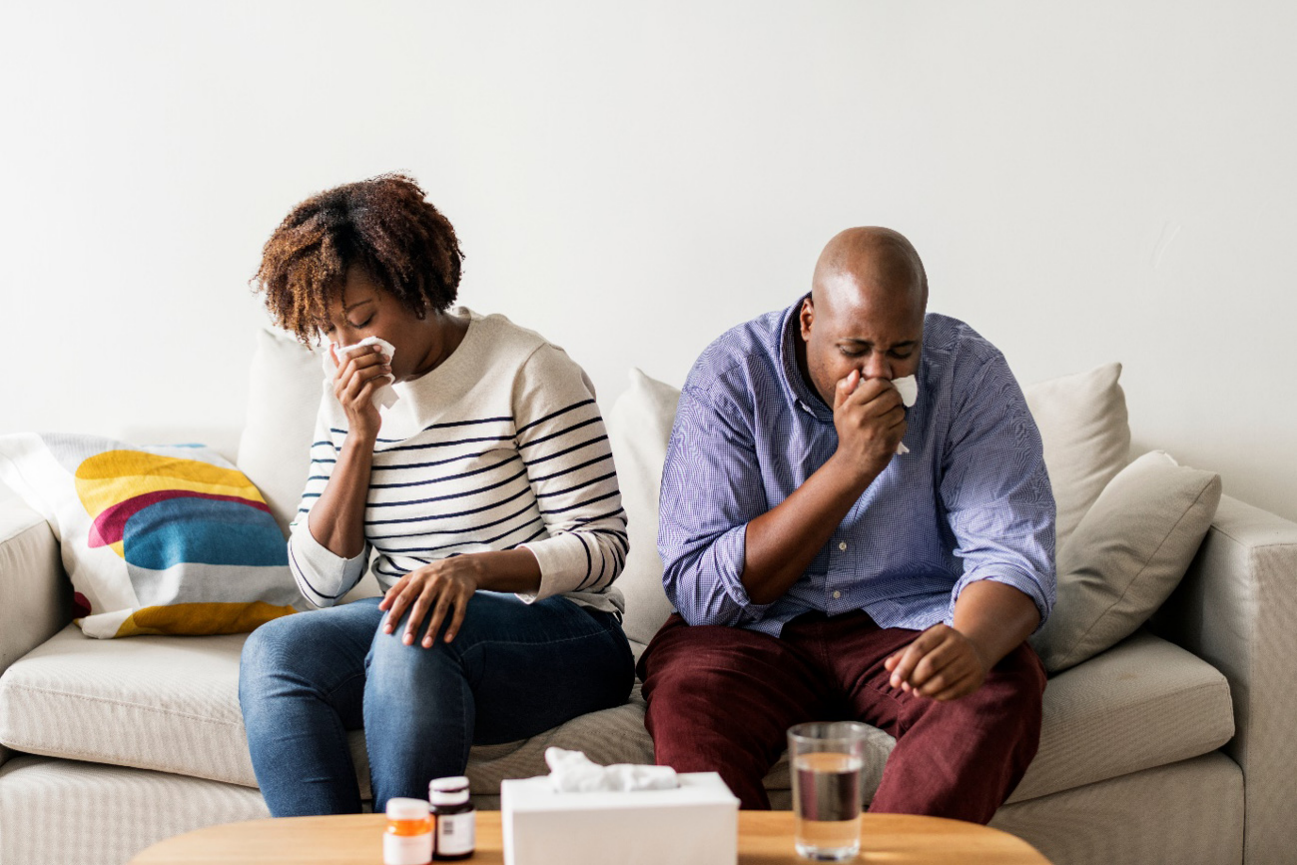

In the UK, low vaccine uptake in the 18–65 age group is a difficult but very important issue. This missed subgroup is important because it includes just over 6 million people who are at-risk due to associated comorbidities. A common concern raised by those who avoid the vaccine is related to its efficacy, due to the variety of strains that may at any time be present. The current UK vaccination programme takes an age-related approach: individuals older than 65 years receive the adjuvanted trivalent vaccine and children aged between 2 and 9 years receive the live attenuated influenza vaccine as a nasal spray; for both groups the vaccine is free. The nasal delivery is highly efficient in delivering vaccine, more so even than injection. The nasal spray contains virus which has been disabled, prepping the immune system without the danger of real infection. This safe and easy way of delivering the vaccine eases uptake for children who may fear needles. As previously mentioned, some pilot studies across the UK offer vaccination to all primary school children. A tailored approach to vaccine delivery is essential. The trivalent and quadrivalent vaccines provide protection against three or four strains respectively. Large outbreaks of unexpected strains are rare but, nonetheless, continuous monitoring ensures the most likely outbreaks are accounted for.

However, it is undeniable that vaccine effectiveness needs improvement. Indeed, any future effort to meet the WHO’s suggested vaccination uptake of 75% must be combined with better vaccines. Vaccine production must also be improved to ensure supply is ready to meet demand. Brexit-related supply roadblocks at Calais, France, such as those raised by Sanofi, who provide 48% of Britain’s vaccine, are a concern [7]. Regardless, vaccination using currently available preparations, saves England £825 429 by reducing visits to the general practitioner, and saves £89 240 781 from costs associated with premature deaths [8].

Apart from individuals with specific allergies and the very young, there should be few barriers to influenza vaccine access. Consider the increased potency of the winter influenza vaccine messaging if it were accompanied by further general subsidisation. Many workplaces, aware of the potential of lost work hours, provide such subsidisation. Bringing the cost down from what may be up to £20 may further encourage members of the public to access the vaccine. Combined with stronger messaging towards the public on the combined responsibility to help protect more vulnerable people and more confident messaging on safety, we can herd society towards fewer debilitating influenza outbreaks.
